# LINKING SELF-REPORTED FATIGUE TO OBJECTIVELY MEASURED PHYSICAL ACTIVITY IN MEN WITH AND WITHOUT HIV

**DOI:** 10.1093/geroni/igad104.1173

**Published:** 2023-12-21

**Authors:** Amal Wanigatunga, Sohyeon Kwon, Frank Palella, Susan Koletar, Stephen Gange, Todd Brown, Joseph Margolick, Jennifer Schrack

**Affiliations:** Johns Hopkins School of Public Health, Baltimore, Maryland, United States; Johns Hopkins School of Public Health, Baltimore, Maryland, United States; Northwestern Feinberg School of Medicine, Chicago, Illinois, United States; Ohio State University School of Medicine, Columbus, Ohio, United States; Johns Hopkins School of Public Health, Baltimore, Maryland, United States; Johns Hopkins School of Medicine, Baltimore, Maryland, United States; Johns Hopkins School of Public Health, Baltimore, Maryland, United States; Johns Hopkins University, Baltimore, Maryland, United States

## Abstract

Fatigue, a symptom commonly associated with reduced physical activity, is difficult to measure among people living with HIV (PLWH), a population with the potential for comorbidity. We examined the association between self-reported fatigue and objectively measured physical activity by HIV serostatus across three commonly used fatigue measures. Self-reported fatigue and accelerometry data were collected cross-sectionally from 699 men (mean age 59 years; 57% PLWH) enrolled in the Multicenter AIDS Cohort Study. Linear regression was used to estimate differences in total daily physical activity (min/day) by self-reported fatigue measured by three separate assessments: 1) “unable to get going”; 2) “felt everything was an effort”; and 3) “work/activity difficulty due to physical health”. Models adjusted for age, BMI (kg/m2), and HIV serostatus, and interactions between HIV serostatus and each fatigue assessment were tested. Fatigue prevalence, defined as “unable to get going” (43%), “felt everything was an effort” (39%), or “work/activity difficulty” (20%) and total daily physical activity (7.4 hours active/d) appeared similar by HIV status. Men reporting “unable to get going” and “work/activity difficulty” were estimated to have an adjusted average of 21 (SE=8) fewer active min/day (p=0.01) and 35 (SE=10) fewer active min/day (p=0.001) than those without not reporting either, respectively. There was an interaction between HIV+ serostatus and “work/activity difficulty” (interaction beta: -54 active minutes/day, p=0.003). No association was detected with “felt everything was an effort” (p=0.16). Among the three questions assessed, “work/activity difficulty” was identified as a potentially useful measure of physical activity limitation in men living with HIV.

